# Glucocorticoid (dexamethasone)-induced metabolome changes in healthy males suggest prediction of response and side effects

**DOI:** 10.1038/srep15954

**Published:** 2015-11-03

**Authors:** Natalie Bordag, Sebastian Klie, Kathrin Jürchott, Janine Vierheller, Hajo Schiewe, Valerie Albrecht, Jörg-Christian Tonn, Christoph Schwartz, Christian Schichor, Joachim Selbig

**Affiliations:** 1 metanomics GmbH, Tegeler Weg 33, 10589 Berlin, Germany; 2 Max Planck Institute of Molecular Plant Physiology, Am Mühlenberg 1, 14476 Potsdam, Germany; 3 Institute for Biochemistry and Biology, University of Potsdam, Karl-Liebknecht-Str. 24-25, 14476 Potsdam, Germany; 4 Metanomics Health GmbH, Tegeler Weg 33, 10589 Berlin, Germany; 5 Department of Neurosurgery, Klinikum Grosshadern, Ludwig-Maximilians-University Munich, Marchioninistr.15, 81377 Munich, Germany

## Abstract

Glucocorticoids are indispensable anti-inflammatory and decongestant drugs with high prevalence of use at ^~^0.9% of the adult population. Better holistic insights into glucocorticoid-induced changes are crucial for effective use as concurrent medication and management of adverse effects. The profiles of 214 metabolites from plasma of 20 male healthy volunteers were recorded prior to and after ingestion of a single dose of 4 mg dexamethasone (+20 mg pantoprazole). Samples were drawn at three predefined time points per day: seven untreated (day 1 midday - day 3 midday) and four treated (day 3 evening - day 4 evening) per volunteer. Statistical analysis revealed tremendous impact of dexamethasone on the metabolome with 150 of 214 metabolites being significantly deregulated on at least one time point after treatment (ANOVA, Benjamini-Hochberg corrected, q < 0.05). Inter-person variability was high and remained uninfluenced by treatment. The clearly visible circadian rhythm prior to treatment was almost completely suppressed and deregulated by dexamethasone. The results draw a holistic picture of the severe metabolic deregulation induced by single-dose, short-term glucocorticoid application. The observed metabolic changes suggest a potential for early detection of severe side effects, raising hope for personalized early countermeasures increasing quality of life and reducing health care costs.

Glucocorticoids, such as the here investigated dexamethasone (DEX), are highly effective anti-inflammatory, immunosuppressant and decongestant drugs. Since decades they have been applied in a variety of disease conditions, e.g., auto-immune diseases, allergic reactions and cancer, often improving quality of life and prognosis immensely. The importance of oral corticosteroids (glucocorticoids and mineralocorticoids) is demonstrated by their wide application, e.g., roughly 0.9% of all adults used oral corticosteroids at any given time point in the UK^1^. Unfortunately, glucocorticoid therapy causes serious systemic side effects, especially in chronic or high dose application, significantly decreases quality of life, life expectancy and increases health care costs^2^,^3^. Adverse effects range from increased sensitivity to stomach acid, adrenal gland depression, immunosuppression, hypertension, psychological disturbances, exogenous Cushing’s syndrome with thin, fragile skin, osteoporosis, muscle atrophy, weight gain and steroid diabetes^4^.

The adverse effects of corticosteroids are inherent to the mode of actions itself, namely activation of the endogenous hormone signalling pathways of natural stress responses in fight-or-flight conditions^5^. Treatment response versus side effect ratios vary considerably depending on the treated individual, applied corticosteroid and time or route of administration. Although practical guides have been published to optimize management of treatment and adverse effects^6^, the suggested monitoring is complicated, time consuming, costly and often not sufficiently performed^1^,^7^. Accordingly, a call for sparing therapies has been repeatedly made^3^, but development of new therapies is slow so that oral corticosteroids will remain irreplaceable for the foreseeable future. A faster approach to alleviate patients’ stress, increase treatment success and reduce health care costs would be the development of easily accessible and understandable biomarkers for the occurrence of side effects.

Another present topic concerning glucocorticoids is the use as concurrent medication, for example in treatment of cancers like glioblastoma. Although glucocorticoids are not curative in cancer conditions, they are often vital for patients, considerably improving severe tumour-related symptoms. However, whether and under which circumstances glucocorticoids enhance or impede radio- and chemotherapies is not finally clarified. On the one hand, glucocorticoid-induced inhibition of respiration can result in increased tumour oxygenation and consequently enhanced tumour radio sensitivity^8^. On the other hand, DEX can interact with drug-induced apoptosis pathways, initiating survival programs and protecting cells against metabolic stress-induced cell death^9^,^10^. DEX can reduce the efficacy of chemotherapy in glioma cells^11^, but this seems to be cell-type-specific^12^. While therapy enhancement is favourable, it would be fatal if a curative therapy has been compromised by concurrent glucocorticoid administration. Thus, more mechanistic and holistic insights into the metabolic impact of this class of drugs are needed.

Despite the long standing application of glucocorticoids, numerous studies about selected metabolites left important questions unanswered, whereas a holistic approach to understand glucocorticoid-induced metabolic changes is not available up to now. Here, we present for the first time, a comprehensive picture of DEX-induced metabolic changes after a single-dose application in healthy, male volunteers.

## Methods

### Study Design

**Figure 1:**
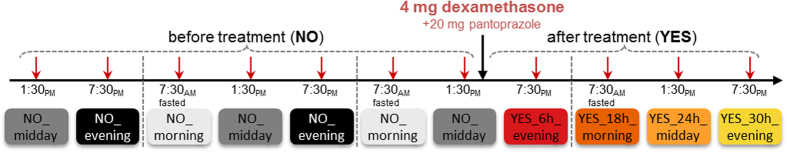
Study design for 20 healthy male volunteers. Naming and colour coding of samples for multivariate and ANOVA plots.

Approval was obtained from the relevant institutional ethical review committee of the Ludwig-Maximilians-University Munich chaired by Prof. Dr. W. Eisenmenger (votum AZ 272-09). The study was performed in accordance with the institutional ethical guidelines and the German drug law (Arzneimittelgesetz, AMG). All enrolled volunteers gave informed consent. Only volunteers without any known chronic or acute disease not taking any medication were included in the study. Human blood plasma samples from 20 healthy, male volunteers (age = 25.5 ± 2.9, body mass index BMI = 23.3 ± 2.7, [mean ± standard deviation SD]) were analysed. Samples were collected for four consecutive days at three time points (morning 7:30 am, midday 1:30 pm, evening 7:30 pm) to account for natural circadian fluctuations. After midday at day 3, the volunteers received a single oral dose of 4 mg dexamethasone (with 20 mg pantoprazole for gastric protection), so that per test person seven untreated (day 1 midday, evening; day 2 morning, midday, evening; day 3 morning, midday) and four treated (day 3 evening; day 4 morning, midday, evening) plasma samples were taken (Fig. 1). For sampling blood was drawn by venous puncture into 7.5 ml K3 EDTA S-Monovette® tubes and centrifuged for 15 min at 1500 g at 4 °C within 30 min after withdrawal. Plasma was cautiously transferred into standard sample tubes, shock frozen in liquid nitrogen within 1 h after withdrawal and stored at −80 °C. Samples were transported on dry ice for metabolomics analysis.

### Metabolic profiling (metabolomics)

Three types of mass spectrometry (MS) analyses were applied to all samples, the semi-quantitative MxP® Broad Profiling and the quantitative MxP® Catecholamines and MxP® Steroids, at metanomics GmbH.

For MxP® Broad Profiling gas chromatography (GC) MS (6890 GC (Agilent) coupled to an 5973 MS-System (Agilent) and liquid chromatography (LC) MS/MS (1100 high performance LC (HPLC) (Agilent) coupled to an API4000 MS/MS-System (Applied Biosystems)) were used as previously described in detail^13^. In short, proteins were removed from plasma samples (60 μL) and subsequently spin filtrated (Ultrafree®-MC 5.0 μm, Milipore) in 5 min. Filtrates were diluted with water, extracted with dichloromethane/ethanol (2:1) and phase separation was achieved by centrifugation for 5 min at 12000 rpm. Subsequently, polar and nonpolar fractions were separated. For LC-MS/MS analyses, both fractions were dried under reduced pressure and reconstituted in appropriate elution solvents. HPLC was performed by gradient elution using methanol/water/formic acid on reversed-phase separation columns. Mass spectrometric detection technology was applied as described in the patent US7196323^14^, which allows MRM in parallel to a full screen analysis. For GC-MS analyses, the non-polar fraction was treated with methanol under acidic conditions to yield the fatty acid methyl esters derived from both free fatty acids and hydrolysed complex lipids. The polar and non-polar fractions were further derivatized with O-methyl-hydroxyamine hydrochloride (20 mg/ml in pyridine) to convert oxo-groups to O-methyloximes and subsequently with a silylating agent (N-Methyl-N-(trimethylsilyl) trifluoroacetamide) before GC-MS analysis. Data were normalized to the median of reference samples which were derived from a pool formed from aliquots of all samples to account for inter- and intra-instrumental variation.

For the quantitative MxP® Catecholamines and MxP® Steroids online SPE-LC-MS/MS (solid phase extraction-LC-MS/MS; Symbiosis Pharma (Spark, Emmen, Netherlands) coupled to an Applied Biosystems API4000 MS/MS-System) was applied to determine quantitative catecholamine and steroid levels as described^15^. The limit of detection or quantification was determined from the daily calibration curve and absolute quantification was performed using stable isotope-labelled standards. Details on nomenclature are available^16^, but in short, the term “*additional*”, abbreviated “*add.*”, was applied to indicate that quantitation can be disturbed by metabolites exhibiting identical analytical characteristics with respect to the quantitation method.

In our study, 201 metabolites achieved sufficient high quality for relative quantification and 13 metabolites for absolute quantification and values can be found as Supplementary Table S1 online.

### Univariate Statistical Analysis (ANOVA)

Metabolite values were log_10_-transformed for statistical analysis to better match normal distribution. A mixed-effects analysis of variance (ANOVA) model (statistical software R 2.8.1^17^, package nlme^18^) was used to determine DEX treatment-induced changes in metabolite abundance. For a range of metabolites diurnal fluctuations were observed, so that the metabolic impact of DEX treatment was only evaluated for matched time points (e.g., morning treated versus morning untreated). To sharpen focus on the DEX-induced effects all samples from corresponding untreated time points (e.g., day one evening, day two evening) were combined. The combination was possible because the number of significantly changed metabolites between two similar untreated time points was always below or close to the expected false-positive rate (around 5% ~11 metabolites for p < 0.05, for naming and colour coding see Fig. 1). The ANOVA model was set up with the categorical fixed factor treatment, the numeric fixed factors age and BMI and the random factor individual (readouts: ratios, p-values, t-values, Benjamini-Hochberg corrected q-values can be found as Supplementary Table S2 online). The ratio is the mean of all samples for one metabolite within one group divided by the mean of all samples for the same metabolite from the reference group. No metabolites correlated significantly with BMI or age (q < 0.1).

### Multivariate Statistical Analysis (PCA, PLS-DA, STATIS)

To obtain a more robust multivariate model, data were further centered and scaled to unit variance. This introduces a common scale for all metabolites independent of their absolute variance, so that models cannot be dominated by few high-variance metabolites.

PCA (principal component analysis) and PLS-DA (partial least squares discriminant analysis) were performed with Simca P+ software (version 12, Umetrics AB, Umea, Sweden). For PLS-DA model performance was quantified by Q^2^ values from leave-one-subject-out cross validation to account for relations between samples from the same volunteer.

STATIS (Structuration des Tableaux A Trois Indices de la Statistique) was developed by Hermier des Plantes and Thiébaut^19^,^20^ and performed here using the statistical software R^17^ with packages ade4^21^ and mixOmics^22^ as previously described with plant metabolic profiles^23^. STATIS is based on the integration of a given set of data tables into an optimum weighted average, called a compromise, which captures what is common to all or a subset of analysed tables.

## Results and Discussion

Blood samples were drawn from 20 healthy male volunteers on four consecutive days (see Fig. 1) in the morning (7:30 am), at midday (1:30 pm) and in the evening (7:30 pm) except for day 1 from which only midday and evening samples were taken. After midday at day 3, the volunteers received a single oral dose of 4 mg dexamethasone (with 20 mg pantoprazole for gastric protection). Metabolite profiling was performed on these 220 blood samples yielding 214 metabolites with 161 unique known metabolites; 22 metabolites included minor levels of other metabolites with identical analytical characteristics, e.g., cysteine (additional: cystine), and 31 unknown metabolites. The study concentrated on a well-defined population sub-group in order to reduce intra-individual variability and complexity sufficiently to allow detection of finer metabolic changes with robust statistical significance.

Pantoprazole is a widely prescribed proton pump inhibitor to treat reflux diseases or to counteract gastrointestinal side-effects of other medications such as glucocorticoid treatment. Pantoprazole specifically and covalently binds to stomach H^+^/K^+^-ATPase thus increasing the stomach pH. Full extent of pH increase is reached after one to several days^24^. The plasma half-life is short with about 2 h, but effect half-life is long with about 24 h because new pumps must be expressed. The impact of pantoprazole on the metabolome is expected to be minimal and is overlaid by dexamethasone effects in this short-term study. However, detailed investigation is recommendable in long-term studies because the elevated pH might impact the gut microbiome and thus nutrient uptake.

### Diurnal fluctuations of plasma metabolite levels

Metabolite levels are well known to exhibit circadian rhythmicity^25^,^26^. The impact of day time was here determined with ANOVA by comparing untreated midday or evening to morning levels. Metabolite levels were found to only monotonously increase or monotonously decrease with progressing time of the day with overall more metabolites increasing than decreasing (see Supplementary Table S2 online).

In total, 10 metabolites were significantly (q < 0.05) decreased, both on midday and evening, 7 only on midday and 8 only in the evening. As expected^27^, steroid production was highest in the morning and levels decreased markedly with progressing time of the day. The strongest decreases were found for corticosterone and cortisol, dropping to 16% and 34%, respectively, of the morning levels. In contrast, 58 metabolites were significantly (q < 0.05) increased both on midday and evening, 7 only on midday and 13 only in the evening. The increase of lipidic metabolites such as TAGs, sphingosines, fatty acids and complex lipid sum parameters, e.g., glycerol, myo-inositol, or galactose moieties from the lipid fraction (released by hydrolysis of complex lipids during sample preparation), some amino acids and related metabolites, some glycolysis intermediates and one xenobiotica (caffeine) towards the evening can be well attributed to common nutrition patterns.

In sum, almost half of the measured metabolome exhibits time of the day-dependent levels and emphasizes importance of time of the day-specific sampling and comparisons.

### DEX highly impacts metabolome

The single-dose DEX treatment had an immediate, long-lasting and overall very strong impact on the metabolome. In total, 150 metabolites were changed significantly (q < 0.05) at any of the time points after treatment. At each time point after treatment between 67 to 79 metabolites were significantly changed but only 9 metabolites were significantly changed in all four time points after treatment (see Supplementary Table S2 online). Seemingly, the single-dose DEX does not invoke a straight forward, unidirectional metabolome response, which then subsides with time, but rather a complex interplay of different mechanisms is activated depending considerably on elapsed time after treatment.

PCA is an undirected method searching for a projection describing as much as possible of the variability in the first component thereby revealing the strongest impact factor. As can be expected for human studies the inter-volunteer variability^28^, i.e., the variation between different volunteers, dominated the first components strongly obscuring treatment effects (see Supplementary Fig. S1 online). In contrast, PLS-DA is directed towards searching the highest difference between *a priori*-defined groups, here treated versus untreated. PLS-DA yielded with Q^2^(cum2) = 63.3% a significant separation of untreated from treated samples along the first two components (see Supplementary Fig. S1). However, as groups are defined *a priori* other impact factors could be overlooked.

**Figure 2:**
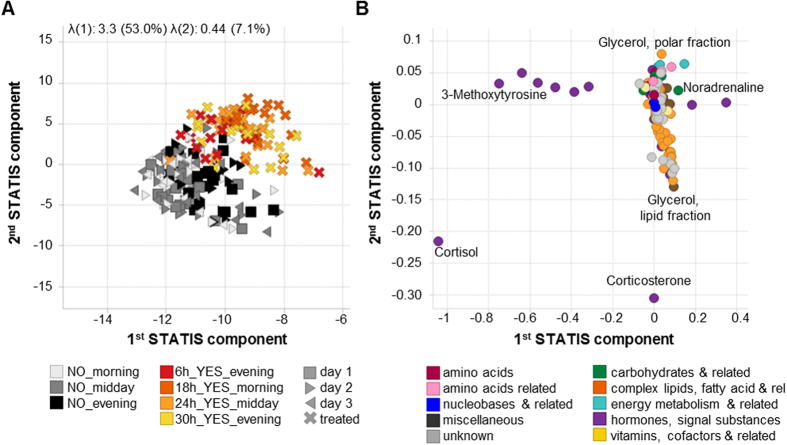
Multivariate Analysis with STATIS. (**A**) Scores plot, showing the contribution of the observations (i.e., samples) according to the undirected STATIS analysis achieving a to PLS-DA comparable separation of untreated vs. treated. (**B**) Corresponding loadings plot, showing the contributions of the variables (i. e., metabolites) according to STATIS.

Therefore also STATIS, which showed promising results for plant metabolomics^23^, was tested. STATIS can investigate multiple observation tables simultaneously and thus reduce variability of low interest (e.g., inter-volunteer) sharpening undirected, multivariate results^23^,^29^. In short, STATIS calculated covariance matrices between the metabolite tables from the different time points for each volunteer, e.g., calculating how similar the metabolic profiles at any two given different time points were for the same volunteer. Based on these 20 covariance matrices (one per volunteer) the compromise of the volunteers was calculated. For the compromise the similarity between volunteer covariance matrices was calculated and volunteers’ covariance matrices were weighed accordingly against each other with higher weights for volunteers more similar to all other and lower weights for dissimilar volunteers (weights can theoretically range from 0 to 1). These weights already reveal that the obtained data set was fairly homogenous, weight differences were low and ranged from 0.26 for the most similar volunteer 4 to 0.17 for the most dissimilar volunteer 8 (see Supplementary Fig. S2). Finally a PCA was performed on the compromise and the extracted principal components were employed to project the analysed tables. The result is a score plot very similar to a typical PCA but with strongly reduced inter-volunteer variability (Fig. 2A).

STATIS achieved similar good separation as the directed PLS-DA and underlines that treatment is the second strongest impact factor. The single DEX dose induces large scale metabolic changes, detectable even after 30 h where plasma levels are estimated to drop to ~2–6% when assuming 1 h delay for oral uptake and half-life of 5–7 h^30^. The persistent impact of DEX on the metabolome even after almost complete plasma clearance results from DEX acting as a transcription factor and the strong impact emphasizes the general regulatory role of corticosteroids. The loadings plot reveals metabolites mainly driving separation of treated versus untreated samples. The separation is driven by almost all of the measured catecholamines and steroids as well as some complex lipids, fatty acids or related metabolites (Fig. 2B) and was reconfirmed by ANOVA results (Supplementary Table S2 online).

### Suppression of endogenous steroid hormone production and interruption of circadian rhythm

**Figure 3:**
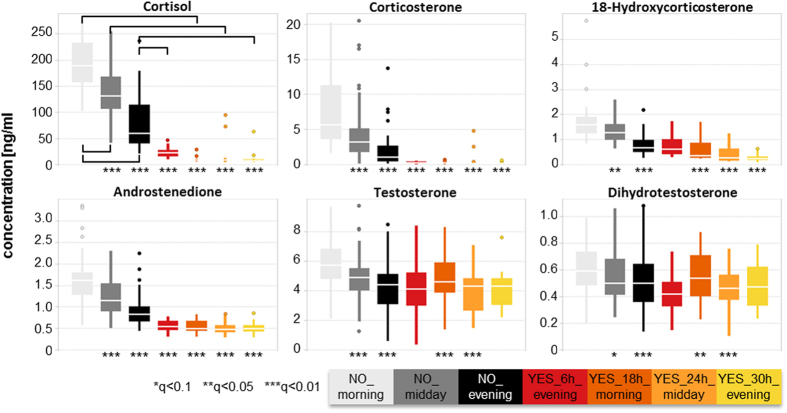
Steroid levels before and after single-dose dexamethasone (DEX). Asterisks mark significance levels as calculated by ANOVA (comparisons as indicated in Cortisol plot), for exact q-values please refer to Supplementary Table S2 online.

Steroid levels show naturally a strong circadian rhythm with highest levels in morning hours, as seen here before treatment (Fig. 3). Steroids are released in short burst and have a fast plasma clearance which explains the high variability observed at untreated time points. Endogenous steroid production is negatively feedback-regulated, lowering adrenal production for higher levels^31^, so that feedback suppression by DEX will occur. Most steroid levels were significantly decreased 6 h after treatment and all were strongly decreased after 18 h and 24 h (Fig. 3). After 30 h levels started to normalize, but were often still significantly lower than before treatment thus persisting despite low estimated plasma levels. Additionally, the circadian rhythm was completely interrupted in the observed first 30 h and presumably also beyond. The suppression was stronger the closer the biosynthetic relationship of the steroid was. The extent of endogenous steroid suppression, both in strength and duration, from a single-dose DEX links well to the side effect adrenal gland depression induced by prolonged glucocorticoid treatment.

Glucocorticoids regulate catecholamine levels^32^ and dexamethasone has been suggested to inhibit catechol-O-methyl transferase (COMT)^33^ as well as to activate monoamine oxidase (MAO)^34^. At first, some decrease was observed which was most pronounced 6 h to 18 h after treatment. Afterwards a pronounced increase followed from 18% to 59% of homovanillic acid (HVA), 3,4-dihydroxyphenylglycol (DOPEG), 3,4-dihydroxyphenylacetic acid (DOPAC) at 24 h and 30 h after treatment (see Supplementary Table S2 online). Catecholamines can be associated with several side effects as they are an integral part of the flight-or-fight response^35^ and have been shown to be involved in the regulation of the immune system^36^. Additionally, changes in plasma levels of HVA are associated with stress^37^, depression^38^ and schizophrenia^39^, although the exact cause-or-effect connection between plasma HVA levels and psychological and behavioural effects^40^ remains to be decoded.

**Figure 4:**
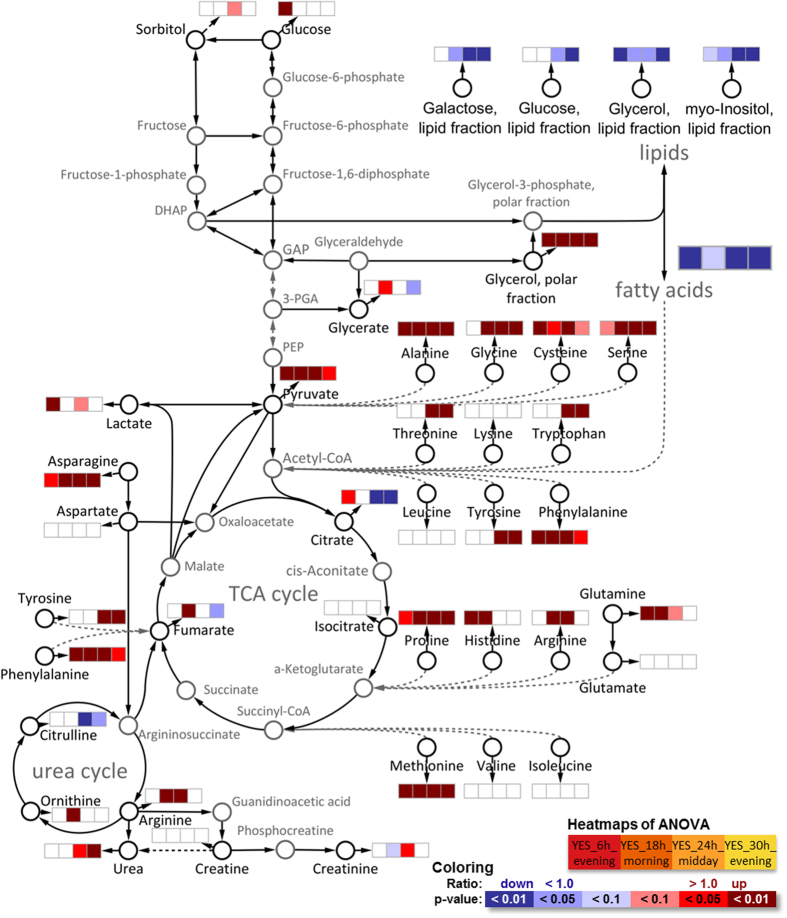
Overview of main energy-generating pathways glycolysis, TCA cycle and urea cycle with connections to amino acids and lipid synthesis/oxidation. Black metabolites were measured and dashed lines implicate several reaction steps which were omitted for the sake of clarity. General trends for lipids and fatty acids are shown and detailed changes are discussed in the following sections.

Simultaneously to the catecholamine increase, their precursor amino acid tyrosine (Fig. 4) was also increased. Tyrosine content in blood is controlled by the activity of the glucocorticoid-dependent enzyme hepatic tyrosine aminotransferase (TAT), a key enzyme of gluconeogenesis^41^. Furthermore a second enzyme, tyrosine hydroxylase, which catalyses the conversion of tyrosine to L-DOPA, the rate limiting step in the synthesis of catecholamines, is increased by glucocorticoids^42^. Tyrosine plasma levels have been proposed as a representative index of glucocorticoid action on metabolism due to their close relationship^43^.

Another connection to psychological side effects could be tryptophan, an essential precursor for serotonin. Tryptophan levels and metabolites of tryptophan metabolites (3-indoxylsulfate, indole-3-acetic acid, indole-3-lactic acid, kynurenic acid) were found to be increased at later time points. Serotonin release and reuptake is effected by acute as well as chronic stressors linked to glucocorticoids^44^. DEX has been shown to indirectly regulate serotonin activity by increasing the expression of the serotonin transporter (5-HTT)^45^ and by inhibiting the biosynthesis through suppression of the expression of tryptophan hydroxylase-2 protein^46^.

### Redisposition of main energy pathways

Glycolysis, tricarboxylic acid (TCA) cycle, urea cycle and their connection with lipids, fatty acids and amino acids can be seen as the main energy pathways. Glucocorticoids regulate energy metabolism and under stress conditions liberate energy substrates by enhancing hepatic gluconeogenesis, reducing glucose utilization, increasing muscle protein catabolism and lipolysis^5^. All these mechanisms were already significantly visible in plasma metabolome after a single-dose DEX (Fig. 4).

The increased gluconeogenesis paired with the reduced glucose utilization were directly visible as 20% increase of plasma glucose 6 h after treatment and the healthy metabolism was able to renormalize glucose levels thereafter. The increased hepatic gluconeogenesis paired with the decreased glucose utilization is discussed as one major mechanism of the side effect insulin resistance and steroid diabetes under chronic glucocorticoid treatment^5^. Additionally, a panel of 10 metabolites was recently found to differ significantly between control subjects and pre-diabetics from 1.5 up to 6 years prior to diabetes diagnosis by Padberg *et al.*^47^ with MxP® Broad Profiling. The panel encompasses 1,5-anhydrosorbitol, 2-hydroxybutyrate, 3-hydroxybutyrate, glucosamine, glucose, glucose-1-phosphate, glyoxylate, lactate, mannosamine, and mannose. All these metabolites were increased in pre-diabetes and reconfirmed in a second study (except 1,5-anhydrosorbitol, which was decreased in study 1 and not reconfirmed in study 2). These metabolites were here similarly increased after DEX treatment (see Supplementary Table S2 online) after 6 h (except 3-hydroxybutyrate after 18 h) and almost all normalized after 24 h to 30 h. These early occurring changes after a single-dose DEX intervention show the sensitivity of the metabolome and link to known DEX side effects before these could clinically manifest (no side effects were observed in our study).

The increase of lipolysis by DEX was well visible from several lipid and fatty acid sum parameters as well as from specific lipid changes. Lipid sum parameters such as “Glycerol, lipid fraction” arise from the hydrolysis of complex lipids with subsequent derivatization for GC analysis and describe the sum of all lipids with such chemical moieties (e.g., glycerol containing lipids). In contrast, the term “Glycerol, polar fraction” originates from free glycerol in plasma, characterizing endogenous glycerol release from glycerol-lipids. Fatty acids represent here the sum of its occurrence both in free and in all lipid-bound forms.

Lipid sum parameters decreased with increasing time after treatment and most became significant 24 h after treatment, except for glycerol, lipid fraction with levels dropping to 41% already 6 h after treatment and remaining lowered to 71% of control levels after 30 h. The liberated glycerol (glycerol, polar fraction) was increased accordingly at all time points after treatment. In agreement with lipid sum parameters most individual lipids such as phosphatidylcholines, lysophosphatidylcholines, triacylglycerols (TAGs) or diacylglycerols (DAGs) were found to be decreased at one or several time points after treatment, except for those with very long (>C20) mono- or polyunsaturated fatty acids (see Supplementary Table S2 online).

The same shift from shorter towards longer fatty acids was observed for fatty acid sum parameters and indicates a differential impact of DEX on mitochondrial beta-oxidation and peroxisomal oxidation pathways of fatty acids. Saturated and mono-unsaturated fatty acids with short (C12) up to long (C20) chain lengths were decreased 6 h, 24 h and 30 h after treatment indicating an increased breakdown of fatty acids for energy generation. Additionally, acylcarnitines mediating transport of fatty acids from the cytosol into the mitochondria during beta-oxidation were increased suggesting a beta-oxidation enhancing effect of DEX. In contrast, very long chained (C22/C24) fatty acids, which are metabolized in the peroxisomes^48^, remained either unchanged or only slightly increased, suggesting low to none impact of DEX on peroxisomal oxidation.

### Modulation of inflammation mediators and precursors

Polyunsaturated fatty acids (PUFAs) can partially act as active signalling molecules and are known precursors for many inflammatory messengers like prostanoids, thromboxanes and leukotrienes^49^,^50^. Here five ω-6 fatty acid namely linoleic acid (LA), γ-linolenic acid (GLA), dihomo-γ-linolenic acid (DGLA), arachidonic acid (ARA) and docosapentaenoic acid (C22:cis[4,7,10,13,16]5) (osbond acid) were measured as well as four ω-3 fatty acids, α-linolenic acid (ALA), eicosapentaenoic acid (EPA), docosapentaenoic acid (C22:cis[7,10,13,16,19]5) (DPA) and docosahexaenoic acid (DHA). All PUFAs decreased with increasing time after treatment becoming significant mostly at 24 h and 30 h (except ARA showing a 6% increase at 18 h, but decreasing afterwards). The observed decrease of PUFAs could either reflect the elevated synthesis of anti-inflammatory messengers or the increased lipolysis or a combination of both. The lower PUFA levels could be a potential additional connection to the increased risk of infection under DEX treatment, should their levels remain decreased under chronic treatment. Additionally, low ω-3 fatty acid levels, as often induced by standard Western diet low in fish, enhance risk for atherosclerosis, hypertension and diabetes^51^.

Sphingomyelins are cell wall components and involved in cell recognition and signalling regulating cell differentiation and apoptosis. Sphingomyelins consist of a ceramide O-linked to a typically charged head group (e.g., choline) and ceramides consist of sphingosine backbone amide-linked typically to a fatty acid. Partially significant trends towards moderately decreased ceramide levels and initially increased sphingosine levels (6 h, 18 h), followed by decreased levels, were observed (see Supplementary Table S2 online). Ceramides act as modulators of metabolism^52^ and high levels were connected with increased inflammation^53^ or increased reactive oxygen species (ROS) levels^54^. Similar to lowered PUFA levels the lower ceramide levels could either reflect the anti-inflammatory effect or the increased lipolysis or a combination of both. The initial increase of sphingosines points rather towards lipolysis activation while the following decrease of sphingosines points towards the anti-inflammatory influence of DEX. *In vitro* studies suggest a connection between sphingolipids and PUFAs. DEX-activated sphingosine kinase 1 (SphK 1) in human fibroblasts increases sphingosine-1-phosphate (S1P) levels, protecting cells from apoptosis^55^. S1P regulates also inflammation and was shown to stimulate ARA release^56^. In addition, ceramide-1-phosphate generated from ceramide by ceramide kinase (CERK) was also identified as crucial factor in the formation of eicosanoid inflammatory mediators (prostaglandins, leukotrienes)^57^.

### Muscle protein and bone metabolism

Glucocorticoids activate muscle proteolysis and inhibit general protein synthesis^58^,^59^, increasing plasma levels of selected amino acids^60^ which was well visible in our results (Fig. 4). Plasma levels of six amino acids (Ala, Met, Asn, Phe, Pro, Ser) were elevated at all four time points. For seven amino acids (His, Trp, Gly, Arg, Thr, Tyr, Glu) levels were only increased at some of these time points. The levels of the other 7 proteinogenic amino acids (Val, Leu, Ile, Lys, Cys, Glu, Asp) were not affected by dexamethasone at any time point. The released amino acids can fuel energy generation, gluconeogenesis or synthesis of upregulated proteins such as acute phase proteins^61^,^62^,^63^. However, the strong protein degradation of skeletal muscle protein in chronic treatments leads to side effects such as skin thinning, weakened connective tissue and muscle atrophy^59^. A possible countermeasure could be a dietary supplementation with limiting amino acids (e.g., Cys, Thr, Ser, Asp, Asn) which improved muscle protein synthesis in polytrauma patients^64^.

Another connection to side effects induced by glucocorticoids is trans-4-hydroxyproline which was increased 24 h and 30 h after treatment. Trans-4-hydroxyproline, resulting from post-translational hydroxylation of proline, is crucial for the stability of collagen and free plasma levels were suggested as marker for bone turnover^65^,^66^. Glucocorticoids suppress bone formation and lead to the development of osteoporosis in long-term treatment^67^,^68^.

### Metabolic phenotypes

**Figure 5:**
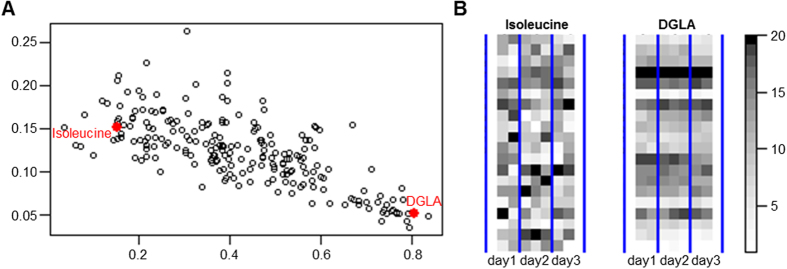
(**A**) Mean versus standard deviation (SD) of pairwise Kendall rank correlations of all untreated time points for each metabolite. (**B**) Colour-gradient coded profiles for DGLA with a stable ranking pattern and isoleucine as example without consistent ranking structure with respect to the individuals.

The metabolic phenotype is determined by an individuals’ genetic make-up^69^ and interplay with environment, remains stable over several healthy years and is sufficiently unique to allow distinguishing between individuals^70^,^71^,^72^. Pairwise Kendall rank correlations for all untreated time points were calculated to identify metabolites with a stable difference between the individual. A set of 27 metabolites with a stable ranking over all untreated time points was identified with *mean(cor)_metabolite_* − *SD(cor)_metabolite_ *≥ 0.6 (Fig. 5). Thereof, 21 were complex lipids, fatty acids and related including all five ω-6 fatty acids, two of four ω-3 fatty acids (EPA, DHA), 8 sphingosines and total cholesterol. These findings are well in line with a genome-wide association study where genetic variants in the fatty acid Δ-5 desaturase (FADS1), a key enzyme in the metabolism of ω-6 and ω-3 fatty acid, were connected to corresponding metabolic phenotypes^69^. The single-dose DEX treatment impacted significantly and markedly levels of most of these 27 metabolites but not the Kendall rank correlations which were similar when time points after treatment were included. Additionally, several of these 27 metabolites showed circadian rhythm dependency, demonstrating how stable metabolic phenotypes are despite different day times or even treatment with DEX.

## Conclusions and Outlook

This study revealed the strong, immediately occurring and long lasting impact of a single-dose short-term application of dexamethasone on numerous biological pathways. The lesser-known but promising method STATIS was able to reduce variability of low interest (e.g., inter-volunteer) and to sharpen results, pinpointing single-dose intervention as the second strongest factor (after inter-volunteer variability). The results were comparable to those from PLS-DA with the advantage that STATIS is an undirected multivariate method without *a priori* bias as potentially introduced by PLS-DA through group definition and in line with univariate ANOVA.

The impact of DEX on the metabolome persisted beyond 30 h after oral intake despite the almost complete plasma clearance after that time. This results from DEX acting as a transcription factor and the impact strength emphasizes the general regulatory role of corticosteroids. To find the endpoint of DEX impact a longer time series is necessary. Additionally, the impact of circadian rhythm on metabolite levels was strongly emphasizing the importance of time of the day-specific sampling and comparisons. Providing circadian rhythm-based glucocorticoid administration already helps to improve therapy and reduce side effects especially in long-term treatment^73^,^74^. A denser time series might allow detection of additional fine regulations. Also, investigating the effects of DEX on the plasma metabolome depending on the time point of administration helps to understand these time-dependent effects which could support further therapy improvements.

Metabolites with stable Kendall ranking of volunteers irrespective of circadian rhythm or treatment were found. These metabolites can be viewed as constituents of the individual-specific metabolic phenotypes. Metabolic phenotypes suggest the possibility of individualized biomarkers potentially discerning between responders and non-responders. However, here observed potentially interesting metabolic phenotypes require substantial validation in follow-up studies because this study concentrated on a narrow population sub-group (healthy, young, normal weight, male) to allow detection of finer metabolic changes in order to pinpoint regions of highest biological interest. The previous study by the SYSTHER consortium showed the strong effect of gender, BMI and age on metabolite levels^28^ so that much larger studies are necessary to establish reliable, valid metabolic phenotypes. Similarly, there might also be differences in the metabolite specific effects of DEX depending on age, BMI or gender although the overall impact strength can be expected to be similarly high.

Many of the observed metabolic changes can be connected to known systemic side effects although no clinical effects or side effects were observed in this study. Systemic side effects occur preferentially and are then especially serious during chronic or high-dose treatments decreasing life expectancy and increasing health care costs^2^,^3^. Shortly, observed changes such as (i) suppression of endogenous steroid production connects to adrenal gland depression, (ii) catecholamine changes relate to psychological problems, (iii) a panel of several carbohydrates and related metabolites links to steroid diabetes, (iv) PUFAs might link to increased risk of infection, atherosclerosis, hypertension and diabetes, (v) increased protein degradation associates to muscle atrophy, and (vi) trans-4-hydroxyproline to osteoporosis. The links are plausibly in line with previous studies on single metabolites or pathways where side effects were clinically manifest.

The severe adverse effects of chronic corticoid treatment make early detection highly desirable to allow for effective countermeasures. Although the relevance and reliability of one single-dose, short-term study is at best explorative, these findings give rise to the idea and hope for early detection of side effects by metabolome changes and could trigger larger studies to develop validated biomarkers. The metabolome shows promising potential for very early detection of adverse developments before manifestation of clinical symptoms thus enabling preventive treatment corrections.

Our results reflect the impressive regulatory role and contribute to a better understanding of glucocorticoid effects helping to develop a more conscious application of this drug.

## Additional Information

**How to cite this article**: Bordag, N. *et al.* Glucocorticoid (dexamethasone)-induced metabolome changes in healthy males suggest prediction of response and side effects. *Sci. Rep.* **5**, 15954; doi: 10.1038/srep15954 (2015).

## Supplementary Material

Supplementary Figures S1 and S2

Supplementary Table S1 and S2
