# Supplementary material for: Glucocorticoid (dexamethasone)-induced metabolome changes in healthy males suggest prediction of response and side effects
Source: Sci Rep. 2015 Nov 3;5:15954. doi: 10.1038/srep15954 (PMC4630650; doi:10.1038/srep15954)
Supplement: Supplementary Figures S1 and S2 [file srep15954-s1.doc]

**Glucocorticoid (dexamethasone)-induced metabolome changes in healthy males suggest prediction of response and side effects**

**Natalie Bordag^1^, Sebastian Klie^2,†^, Kathrin Jürchott^3^, Janine Vierheller^3^, Hajo Schiewe^4^,**

**Valerie Albrecht^5^, Jörg-Christian Tonn^5^, Christoph Schwartz^5^, Christian Schichor^5,♯^ &**

**Joachim Selbig^3,♯^**

^1^metanomics GmbH, Tegeler Weg 33, 10589 Berlin, Germany. ^2^Max Planck Institute of Molecular Plant Physiology,

Am Mühlenberg 1, 14476 Potsdam, Germany. ^3^Institute for Biochemistry and Biology, University of Potsdam,

Karl-Liebknecht-Str. 24-25, 14476 Potsdam, Germany. ^4^Metanomics Health GmbH, Tegeler Weg 33, 10589

Berlin, Germany. ^5^Department of Neurosurgery, Klinikum Grosshadern, Ludwig-Maximilians-University Munich,

Marchioninistr.15, 81377 Munich, Germany. ^†^Present address: Targenomix GmbH, Am Mühlenberg 11, 14476

Potsdam, Germany. ^♯^Shared senior authors. Correspondence and requests for materials should be addressed to

N.B. (email: natalie@bordag.com)

| 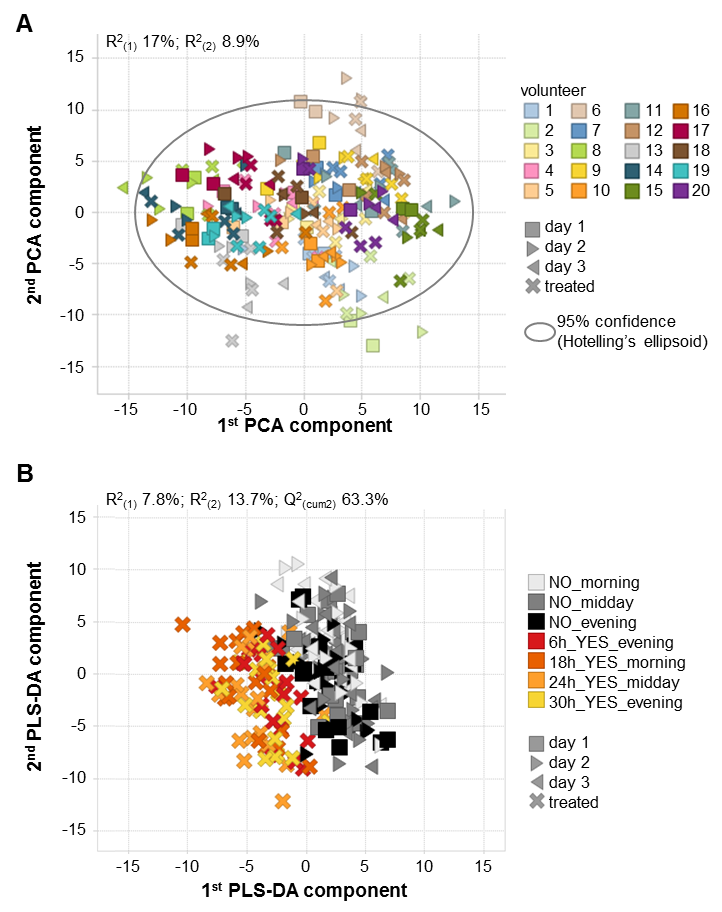 |
| --- |
| Supplementary Figure S1: PCA and PLS-DA scores plot. A PCA scores plot coloured by volunteer number. Inter-volunteer, i.e., the variation between different volunteers, dominated the first components obscuring treatment effects. B Scores plot of directed PLS-DA showing a significant separation of untreated from treated samples. Cross-validation was performed by the leave-one-subject-out method to account for relations between samples from the same volunteer. |

|  |
| --- |
| Supplementary Figure S2: STATIS plot of Rv2 coefficient versus table weights showing the similarity structure of volunteers, with volunteer 4 being most similar and volunteer 8 most dissimilar. |
